# Clinical Impact of Endoscopic Submucosal Dissection for Gastric Neuroendocrine Tumors: A Retrospective Study from Mainland of China

**DOI:** 10.1100/2012/869769

**Published:** 2012-12-23

**Authors:** Wei-Feng Chen, Ping-Hong Zhou, Quan-Lin Li, Mei-Dong Xu, Li-Qing Yao

**Affiliations:** Endoscopy Center and Endoscopy Research Institute, Zhongshan Hospital, Fudan University, 180 FengLin Road, Shanghai, Xuhui 200032, China

## Abstract

As a minimally invasive technique, endoscopic resection may benefit patients diagnosed with early stage gastrointestinal neuroendocrine tumors (NETs). However, no studies have yet been published in which endoscopic submucosal dissection (ESD) has been applied for gastric NETs. For the first time a research group in China applied ESD to remove gastric NETs, and indicated that ESD should be considered for treatment of eligible gastric NETs because the technique shows a high histologically complete resection rate, provides accurate histopathological evaluation, has a low complication rate, and can be performed within a reasonable timeframe.

## 1. Introduction

Gastric NETs have been previously thought to be extremely rare lesions. However, over the last 50 years, the incidence of gastric neuroendocrine tumors (NETs) has been increasing in most countries because of better awareness and an increased widespread use of upper gastrointestinal endoscopy [1]. In the preendoscopic era, they comprised only 0.3% of all gastric tumors and 1.9% of all gastrointestinal NETs. More recent studies have shown that as many as 10–30% of all NETs may occur in the stomach [1]. Nowadays, more and more gastric NETs are usually diagnosed at an early stage (tumor size < 11–20 mm and limited to the mucosa/submucosa) [2] and thus can be managed with local excision including endoscopic treatment because of a low frequency of lymph node and distant metastasis. As a minimally invasive technique, endoscopic resection may benefit patients diagnosed with gastric NETs. It offers the promise of localized treatment of these tumors, with relatively few complications and low mortality.

Various endoscopic resection procedures such as endoscopic polypectomy, strip biopsy, aspiration resection, and band-snare resection have been described as potential treatment procedures for gastric NETs [3–5]. However, complete resection of NETs is difficult with conventional polypectomy and endoscopic mucosal resection (EMR) because most gastrointestinal NETs are not confined to the mucosa but, rather, invade the submucosa [6], which results in frequent involvement of the resection margin. Polypectomy may not provide adequate resection margins, and additional surgical intervention may be needed.

Endoscopic submucosal dissection (ESD) is a method of endoscopic resection and has the advantage of a high probability of en bloc and histologically complete resection even in submucosal tumors because the technique involves dissection of the submucosal tissue beneath the lesion [7]. To date, the fact that ESD can facilitate histologically complete resection of NETs has been verified on the use of ESD for treatment of rectal NETs [8–10]. However, limited systematic studies in which ESD has been applied for gastric NETs have been published. The purpose of this paper was to provide a better understanding of the endoscopic features of these tumors and to retrospectively evaluate the clinical impact of ESD for gastric NETs.

## 2. Patients and Methods

### 2.1. Patients

With the approval of the institutional review board, from January 2008 to January 2012, 25 patients with confirmed histological diagnosis of gastric neuroendocrine neoplasms were treated with ESD. None had regional lymph node enlargement and distant metastases to the liver or lung on computerized tomography (CT) scanning or endoscopic ultrasonography (EUS) before ESD. Tumor characteristics, complete resection rate, complications, local recurrences, and distant metastases were evaluated in all patients. Informed patient's consent was obtained prior to the procedures.

### 2.2. ESD Procedures

Preoperative EUS (high-frequency miniprobe, UM-2R, 12 MHz; UM-3R, 20 MHz, Olympus) was performed to evaluate the depth of tumor invasion and the involvement of regional lymph nodes. The existence of lymph node and distant metastasis was surveyed by contrast-enhanced CT, abdomen ultrasound, and chest X-ray.

To dissect the tumor, endoscopic submucosal dissection was attempted with a single-channel gastroscope (GIF-H260, Olympus) and an insulated-tip electrosurgical knife (KD-611L, Olympus) or hook knife (KD-620LR, Olympus). A transparent cap (D-201-11304, Olympus) was attached to the tip of the gastroscope to provide direct views of the submucosal layer. Other equipment included injection needle (NM-4L-1, Olympus), grasping forceps (FG-8U-1, Olympus), snare (SD-230U-20, Olympus), hot biopsy forceps (FD-410LR, Olympus), clips (HX-610-90, HX-600-135, Olympus), high-frequency generator (ICC-200, ERBE), and argon plasma coagulation unit (APC300, ERBE).

**Figure 1:**
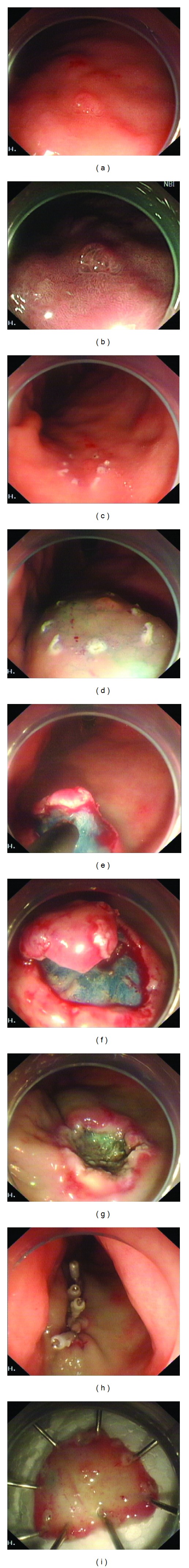
The procedure of endoscopic submucosal dissection for a gastric neuroendocrine tumor. (a, b) A sessile polyp with a reddened surface of the gastric body. (c) Making. (d) Injection. (e, f) Dissection. (g) Resultant artificial ulcer. (h) Closure of the defect with metallic clips. (i) Completely resected specimen.

Patients were treated under general anesthesia. After making several marking dots with argon plasma coagulation around the lesion, a mixture solution (including 100 mL of normal saline, 1 mL of indigo carmine, and 1 mL of epinephrine) was injected into the submucosa. The mucosa was incised outside the marking dots. Direct dissection of the submucosal layer beneath the tumor was then performed under direct vision to achieve complete en bloc resection of the specimen. The tumor was dissected along the capsule, and saline solution was injected repeatedly during the dissection when necessary. The resultant artificial ulcer was managed routinely with argon plasma coagulation to prevent delayed bleeding, and hemoclips were used to close the deeply dissected areas as needed (Figure 1).

### 2.3. Clinicopathological Categorization and Pathological Evaluation

There is a clinicopathological categorization of the gastric NETs which distinguishes four types of neuroendocrine neoplasms of the stomach [2]: type I is those arising in chronic atrophic gastritis with hypergastrinemia; type II occurs in patients with hypergastrinemia due to the Zollinger-Ellison syndrome in association with multiple endocrine neoplasia type I; type III is gastric NET not associated with any specific pathogenetic background; poorly differentiated neuroendocrine carcinomas are nowadays classified as type IV neuroendocrine neoplasms of the stomach.

The WHO 2010 classification of tumours of the digestive system was used for histopathologic evaluation [11]. Mitotic count per 10 high-power field (HPF) or Ki-67 Index per 400–2000 cells was used for grading and staging. On the basis of proliferative activity, gastric neuroendocrine neoplasms are graded as G1, G2, or G3. Low to intermediate grade tumors (G1-G2) are defined as NETs (previously referred to as carcinoids) whereas high-grade carcinomas (G3) are termed neuroendocrine carcinomas (NECs).

En bloc resection refers to a resection in one piece. A resection with a tumor-free margin in which both the lateral and basal margins were free of tumor cells was considered as a complete resection. A resection in which the tumor extended into the lateral or basal margin, or the margins were indeterminate because of artificial burn effects, was considered as an incomplete resection.

### 2.4. Followup

Patients underwent followup endoscopy and/or EUS at 1, 3, 6, and 12 months after ESD and annually thereafter to view the healing of the wound and to check any tumor residual or recurrence. Close followup by abdomen ultrasound, contrast-enhanced CT, and chest radiography were carried out to evaluate distant metastasis every 6 months. A final checkup was performed via telephone questionnaires in August 2012. At that time, followup data for 100.0% of the patients were available for evaluation.

## 3. Results

Patient characteristics, lesion features, and clinical outcomes are summarized in Table 1. The study cohort consisted of 8 men and 17 women, aged from 35 to 82 years. 4 cases had 2 tumors, and 2 cases had 3 tumors. Of the 33 lesions, 1 of them located in the cardia, 5 in the gastric fundus, 26 in the gastric body, and 1 in the gastric antrum. All lesions were found incidentally during routine upper gastrointestinal endoscopy for other indications such as anemia, reflux symptoms, or nonspecific abdominal symptoms. None had symptoms of carcinoid syndrome. With respect to macroscopic appearance, 12 patients had submucosal tumors with a central depression or erosion on top, 10 patients had sessile polyps with a reddened surface, 2 patients had erosion-type tumors, and 1 patient had a tumor with superficial ulcer.

With respect to clinicopathological categorization, 22 lesions in 15 patients were type I gastric NETs arising in chronic atrophic gastritis with hypergastrinemia, while other 11 lesions in 10 patients were type III because of absence of atrophic gastritis in these cases. None showed metastatic disease to lymph nodes or distal organs on preoperative examinations. Before ESD procedures, histological diagnosis of gastric NETs had been confirmed via biopsies in 4 cases.

All the tumors were removed in an en bloc fashion (33/33, 100%). The average maximum diameter of the lesions was 8.2 mm (range 2–30 mm), and the procedure time was 22.5 minutes (range 10–45 minutes). Results of pathological studies determined that 30 lesions were NET-G1 and 3 lesions were NET-G2. Complete resection was achieved in all the tumors (33/33, 100%). All of them were confined to the submucosa in histopathologic assessment, and no lymphovascular invasion was observed in any of the tumors.

Delayed bleeding occurred in one case 3 days after ESD. Successful hemostasis was achieved by coagulating forceps and spraying with thrombin during emergency endoscopy. The procedure-related perforation was not seen in any tumor.

Because type III gastric NETs with diameter larger than 10 mm may have high risks of metastasis, additional surgical intervention should be considered in 7 cases. However, only 1 of them underwent additional surgery, and we could not reveal residual lesions or metastatic lymph nodes in the surgical specimens. Other 6 cases refused additional surgery, citing their age, physical condition, or other personal reasons. Therefore, they were under careful followup.

During a mean of 28.9 months (range 7–55 months) followup periods, local recurrence occurred in two patients after initial ESD (case no. 1 and no. 12). Both of them then underwent repeat ESD successfully. Metastasis to lymph nodes or distal organs was not observed in any patient. No patients died during the study period.

## 4. Discussion

Because most gastrointestinal NETs are not confined to the mucosa but, rather, invade the submucosa [6], we, thus, chose ESD for the treatment of gastric NETs in the current study. Most of the lesions extended into the submucosa (75.8%, 25/33), and complete resection was achieved for all the tumors in this study. High histologically complete resection rate of ESD may give several advantages for the treatment of gastric NETs [8–10]. First, histologically complete resection can provide a substantial amount of submucosal tissue and accurate determination of lymphovascular invasion, and histological grading is possible and can inform decisions regarding subsequent therapy. Second, incomplete resection of tumors results in the need for additional surgery, and complete resection allows us to reduce the incidence of unnecessary surgery. Third, repeat endoscopic resection of remnant tumor after an initial incomplete endoscopic resection may be difficult because of fibrosis that prevents lifting of the lesion by submucosal injection. Therefore, we recommend histologically complete resection of gastric NETs even when lesions are small, and the present study indicates that ESD may maximize the likelihood of such an outcome because of complete resection.

Gastric neuroendocrine neoplasms are divided into four groups by the clinicopathological classification: type I: NETs associated with type A chronic gastritis; type II: carcinoids with endocrine neoplasia; type III: sporadic carcinoids without hypergastrinemia; type IV: poorly differentiated neuroendocrine carcinomas [2]. Type I is the most frequent and comprises approximately 65% of all gastric NETs, while Type III is less frequent (21%) [1]. Types I and II gastric NETs frequently show less lymph node involvement compared with type III [2, 12]. Gastric NETs with submucosal invasion and muscularis propria invasion also show high incidences of metastasis [13]. Therefore, the indication for rescue surgery after endoscopic resection is usually based upon the size, type, depth of invasion, grade, and stage of the gastric NET disease [3]. In general, additional surgical intervention is recommended in the case of type I or type II gastric NETs with positive margins, size > 20 mm, G2-G3 histological grading, invasion into the muscularis propria, or vessel infiltration of tumor cells. Additional surgery was also recommended in the case of type III gastric NETs with size > 10 mm irrespective of other risk factors. Surgery was the only treatment of choice in case of a localized type IV gastric NET. According to this, additional surgical intervention should be considered in 7 cases in our study; however, 6 of them refused additional surgery, citing their age, physical condition, or other personal reasons. During a two-year term followup, local recurrence or distal metastasis did not occur in these 6 patients.

Bleeding and perforation are the two main complications of ESD. In this study, only one case had delayed bleeding 3 days after ESD. Successful hemostasis was achieved by coagulating forceps and spraying with thrombin during emergency endoscopy. No patient had immediate or delayed perforation. The relatively low ESD complication rate most likely reflects the small size of lesions. Furthermore, we always keep our minds upon the prevention and handling of bleeding during procedure. Once bleeding occurs during ESD, hemostasis may take a long time, and the endoscopic view may be affected; blind hemostasis may eventually lead to perforation. In our study, immediate minor bleeding was treated successfully by grasping the bleeding vessels with hot biopsy forceps and coagulating them during ESD. Direct coagulation with the hook knife could be done for small vessels in the submucosa, and metallic clips were often deployed for more brisk bleeding.

In conclusion, the present study indicates that because of complete resection, ESD may reasonably serve as radical treatment for gastric NETs when lesions are within the existing criteria. It also provides enough histological information for tumor grading and staging even when lesions are beyond the selection criteria, which informs decisions regarding subsequent surgery. In addition, endoscopic treatment might be also considered in particular in patients with a high risk of perioperative complications due to old age or advanced comorbidity, for example, or if there are other contraindications to major surgery, even though the lesions are little beyond the existing criteria.

## Figures and Tables

**Table 1:** Clinicopathological characteristics of 25 patients with gastric neuroendocrine tumors treated by endoscopic submucosal dissection.

Case no.	Age, y	Gender	Location	Macroscopic appearance	Clinicopathological type	Size, mm	En bloc resection	Depth of invasion	WHO classification	Lateral margin	Vertical margin	Vessel invasion	Complication	Additional surgery	Followup, mo
1	36	F	Gastric body	Submucosal tumor	I	4, 4, 3	Yes	Mucosa	NET-G1	(−)	(−)	Absent	None	None	55
2	41	M	Gastric antrum	Submucosal tumor	III	10	Yes	Submucosa	NET-G1	(−)	(−)	Absent	None	None	52
3	71	M	Gastric body	Submucosal tumor	III	18	Yes	Submucosa	NET-G1	(−)	(−)	Absent	None	Reject	45
4	53	M	Gastric body	Submucosal tumor	I	5	Yes	Submucosa	NET-G1	(−)	(−)	Absent	None	None	44
5	55	F	Gastric body	Submucosal tumor	III	10	Yes	Submucosa	NET-G1	(−)	(−)	Absent	None	None	41
6	82	F	Gastric fundus	Submucosal tumor	III	30	Yes	Submucosa	NET-G2	(−)	(−)	Absent	None	Reject	40
7	35	F	Gastric fundus	Polyp	I	6	Yes	Submucosa	NET-G1	(−)	(−)	Absent	None	None	38
8	40	F	Gastric body	Polyp	I	6	Yes	Submucosa	NET-G1	(−)	(−)	Absent	None	None	35
9	47	F	Gastric body	Submucosal tumor	I	4	Yes	Submucosa	NET-G1	(−)	(−)	Absent	None	None	35
10	56	F	Gastric body	Polyp	I	3, 2	Yes	Mucosa	NET-G1	(−)	(−)	Absent	None	None	31
11	51	F	Gastric body	Submucosal tumor	III	8	Yes	Submucosa	NET-G1	(−)	(−)	Absent	None	None	29
12	55	M	Gastric body	Polyp	I	8, 3, 3	Yes	Submucosa	NET-G1	(−)	(−)	Absent	None	None	28
13	55	M	Gastric body	Polyp	I	4	Yes	Submucosa	NET-G1	(−)	(−)	Absent	None	None	28
14	57	M	Gastric body	Submucosal tumor	III	15	Yes	Submucosa	NET-G1	(−)	(−)	Absent	Delayed bleeding	Reject	26
15	35	F	Gastric body	Submucosal tumor	III	25	Yes	Submucosa	NET-G2	(−)	(−)	Absent	None	Yes	25
16	45	F	Gastric body	Polyp	I	6, 3	Yes	Submucosa	NET-G1	(−)	(−)	Absent	None	None	22
17	48	F	Gastric body	Polyp	I	5	Yes	Submucosa	NET-G1	(−)	(−)	Absent	None	None	21
18	55	M	Gastric body	Polyp	I	5	Yes	Submucosa	NET-G1	(−)	(−)	Absent	None	None	20
19	65	F	Cardia	Submucosal tumor	III	20	Yes	Submucosa	NET-G1	(−)	(−)	Absent	None	Reject	20
20	35	F	Gastric fundus	Erosion	I	5, 3	Yes	Submucosa	NET-G1	(−)	(−)	Absent	None	None	20
21	47	M	Gastric fundus	Polyp	I	4	Yes	Mucosa	NET-G1	(−)	(−)	Absent	None	None	20
22	57	F	Gastric body	Polyp	I	8	Yes	Mucosa	NET-G1	(−)	(−)	Absent	None	None	18
23	52	F	Gastric body	Superficial ulcer	III	15	Yes	Submucosa	NET-G2	(−)	(−)	Absent	None	Reject	12
24	40	F	Gastric body	Submucosal tumor	III	20, 5	Yes	Submucosa	NET-G1	(−)	(−)	Absent	None	Reject	11
25	70	F	Gastric body	Erosion	I	5	Yes	Mucosa	NET-G1	(−)	(−)	Absent	None	None	7

NET: neuroendocrine tumor. Reject: the patient rejected the additional surgery.
